# Impacts of a climate change initiative on air pollutant emissions: Insights from the Covenant of Mayors

**DOI:** 10.1016/j.envint.2020.106029

**Published:** 2020-12

**Authors:** Emanuela Peduzzi, Marta Giulia Baldi, Enrico Pisoni, Albana Kona, Paolo Bertoldi, Fabio Monforti-Ferrario

**Affiliations:** European Commission, Joint Research Centre (JRC), Ispra, Italy

**Keywords:** Covenant of Mayors, Climate change, Air quality, Urban air pollution

## Abstract

The Covenant of Mayors (CoM) is a successful European initiative which encourages local authorities to be proactive in fighting climate change. Recently, it expanded to cover adaptation and energy access/poverty and became a global initiative. In this study we investigate an additional perspective: synergies and trade-offs between climate and air quality. Signatories pledge to reduce their Greenhouse gas (GHG) emissions and voluntarily report their emissions, energy consumption and the measures that they carry out to reach their goals. We develop a methodology to estimate air pollutant emissions corresponding to CO_2_ emissions CoM signatories report, using information they already submit and national estimates of air pollutant emission factors. The methodology is applied to over 1600 signatories in Europe, representing over 80 million inhabitants. Results show that, in general, signatories are reducing both types of emissions. However, there are also cases where emissions increase. We explore the reasons behind these changes and highlight the role of technological improvement. This work calls for an increased coherence between climate and air quality plans at the local scale and provides a first step and a tool to support signatories, even the smallest ones, to move in this direction.

## Highlights

• Evaluation of air pollutant emissions corresponding to locally reported CO_2_ emissions.
• Methodology applied to over 1600 Covenant of Mayors signatories.
• Most changes correspond to co-benefits for both climate and air pollution.
• The role of technological improvement to limit emissions is highlighted.

## 1 Introduction

The Covenant of Mayors (CoM) aims to engage and empower small and large municipalities alike in the reduction of Greenhouse gas (GHG) emissions (Kona et al., 2018, Melica et al., 2018). In August 2019 as many as 9693 signatories (95% in the EU) representing a population of 312.5 M inhabitants (75% in the EU) had joined the CoM (Bertoldi et al., 2019). Signatories pledge to report their CO_2_ emissions and reduce them by 40% by 2030 (initially the target was set to 20% by 2020) (Bertoldi et al., 2018). The actions they put in place inevitably have trade-offs and co-benefits with respect to air pollution, a locally relevant issue. Actions (or measures) range from ‘small’ ones, such as the installation of solar panels in public swimming pools or the creation of a bicycle path, to ‘very large’ ones, such as the entire mobility plan of a city.

Trade-offs and co-benefits between climate change and air pollution are well known (Nemet et al., 2010). They have been evaluated at global scale (West et al., 2013, Shindell et al., 2018, Vandyck et al., 2018), at European scale (Weitzel et al., 2019) and at city scale (Sabel et al., 2016, Perez et al., 2015). These studies rely on modelling to take into account different policy scenarios and evaluate their impacts. Some cities are also integrating energy, climate and air quality planning as for example London (Greater London Authority, 2018) and Barcelona (Ajuntament de Barcelona, 2011-2020). However, not every local authority has the expertise and capability to do so.

A first step to support local authorities in this aspect is to try and extract information from the data they already submit to the CoM. This was done, for the first time, in our previous work (Monforti-Ferrario et al., 2018) where we quantified the impact of energy efficiency measures signatories pledged to implement, i.e. win–win measures that are good for both air quality and climate.

The objective of the present work is to extend the analysis to consider also side-effects on air pollutant emissions and consider, instead of pledged measures, actual progress. The core novelty consists of a methodology to estimate, from the data signatories report in terms of final energy use and CO_2_ emissions, in baseline and monitoring reports, the corresponding emissions of air pollutants. These reports show a ‘snapshot’ of the signatories’ final energy use in two different years, but they are not directly linked to the measures put in place. Therefore, even though we cannot consider single actions using these data, it is possible to evaluate the change in the time interval between baseline and monitoring years. The estimate of air pollutant emissions is obtained by combining CoM data with data obtained from air pollutant emission scenarios or inventories, such as the Greenhouse Gas - Air Pollution Interations and Synergies (GAINS) model (Amann et al., 2011, IIASA, 2020) and the Emission Database for Global Atmospheric Research (EDGAR) (Crippa et al., 2018). The impact of technological improvement is also evaluated by comparing emissions of air pollutants in the monitoring year and in the baseline year considering the evolution of air pollutant emission factors (with technological improvement) and with unchanged air pollutant emissions factors with respect to the baseline year (without technological improvement).

The methodology is applied to over 1600 selected signatories showing that, in most cases, they are reducing both types of emissions. However, it is also possible to identify cases where CO_2_ and/or air pollutant emissions have increased and trade-offs. Results show that reported increase of biomass burning for residential heating, often considered neutral in terms of CO_2_ emissions, can have a large impact on the emissions of air pollutants. On the contrary, the role of technological improvement is shown to be particularly important to limit PM2.5 emissions in the transport sector despite the increases in consumption (in particular for diesel cars).

Finally, this analysis shows that there is the potential to integrate air quality aspects in the CoM, which could guarantee consistency between air quality and climate policies at local scale.

## 2 Methods

Signatories submit a Baseline Emission Inventory (BEI), for a baseline year of their choice, summarising energy consumption and CO_2_ emissions for different sectors and fuels (referred to as *energy carriers*). The actions, implemented and planned to achieve the emission reduction targets, are also reported (note that signatory cities pledge actions to support the implementation of the 40 % greenhouse gas-reduction target by 2030). To monitor the evolution of emission reductions the signatories submit also a Monitoring Emission Inventory (MEI), where they summarise, in the same format as the BEI, the energy consumption and the CO_2_ emissions for the monitoring year. BEI, MEI and actions are described by each signatory in a Sustainable Energy and Climate Action Plan (SECAP). The changes in energy consumption (by sector carrier) between BEI and MEI is what we use in this work.

In addition to the CoM data, we use the GAINS model. GAINS (Amann et al., 2011, IIASA, 2020) is a tool used to support air quality policies at European level. Through its web-interface it is possible to access a wealth of data regarding emissions. For example, it is possible to obtain *activity levels* (e.g. fuel consumption, in PJ) and the corresponding *emissions* of air pollutants (e.g. kton of NO_x_) per *sector* (e.g. passenger cars), *activity* (e.g. gasoline) per country and per different scenarios starting from 1990 to 2030 with 5 years intervals. The ratio between emissions and activities is referred to as *implied emission factor*, which we note as ${∊}_{s,c,y}^{p}$ (per precursor *p*, sub-sector *s*, carrier *c* and year *y*, in kton/PJ for example).

**Fig. 1:**
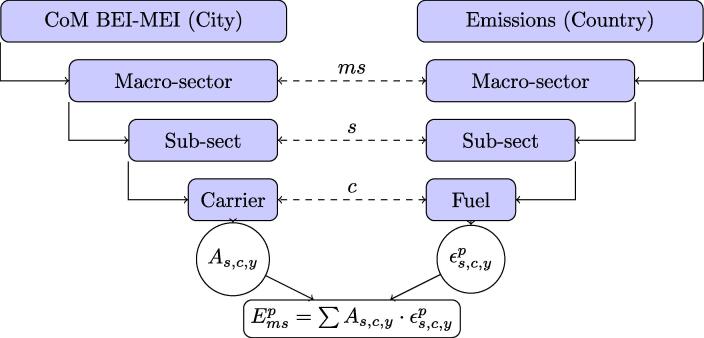
Summary of the methodology to estimate the emission of precursors for each signatory. The methodology combines activity level (*A*) information at the signatory scale from the CoM database and the emission factors (∊) for the corresponding country level from an emission scenario provided by the GAINS model (or by EDGAR). *ms: macro-sector, s: sub-sector, c: carrier, y: year, p: pollutant.*

The results of the present study rely on the matching of the *sectors* (e.g. public transport) and *carriers* (e.g. diesel fuel) as defined by the CoM inventories, to the *sectors* (e.g. buses) and *activities* (e.g. medium distillates), as defined by the GAINS model (or EDGAR). It is therefore possible to associate different (implied) emission factors to the CoM sectors and carriers (and reporting year) and estimate the emissions of air pollutants corresponding to the reported energy consumption. Fig. 1 summarises the approach whereas the following sections explain it in further detail.

### 2.1 Energy consumption by sector and carrier: the CoM database

The CoM data used in the current paper are reported by the signatories in the CoM platform. The reported data must reflect accurately the content of the official SECAP document that each signatory submits. We provide here a brief description of the activity data and emissions reported in the platform and used in the present analysis. We are considering data referring to the residential (RES) and transport (TRA) macro-sectors.

• macro-sector RES (residential) corresponds to ‘Stationary Energy/Buildings’: CO_2_ direct and indirect emissions (due to the consumption of grid-supplied electricity) come from final energy consumption in residential, commercial and institutional buildings and facilities. Emissions from industrial buildings and facilities, agriculture/forestry/fisheries, and from ‘energy generation’ industries are not accounted for in our analysis. Note that heat and cold supplied by district heating/cooling networks are not included.
• macro-sector TRA corresponds to ‘Transportation’: all CO_2_ emissions (direct emission from fuel combustion and indirect emission due to consumption of grid-supplied energy) occurring for road transport within the local authority boundary are reported. Emissions, disaggregated into municipal, public, private and commercial transport, are accounted for in our analysis within the Transport macro-sector.

It should be noted that in the RES sector we are not taking into account emissions, neither CO_2_ nor air pollutants, related to heat and cold supplied by district heating/cooling networks (of the 1653 cities selected for this work, 208 report the use of district heating or cooling in their BEI or MEI). For district heating reporting is not mandatory and therefore data are sparse. The available data-set does not allow us to consistently infer local emissions of air pollutants as we do not know with which technologies and fuels heat and cold are produced. The same problem arises for electricity consumption, but whereas heat and cold are generally produced locally, electricity generation is generally not located within the city boundaries and supplied by the grid. For electricity therefore we do consider CO_2_ emissions (as reported by the signatories) but, as we cannot infer local emissions of air pollutants from a possible local contribution, these pollutant emissions cannot be accounted for at the moment.

CO_2_ emissions reported by the signatories are calculated through default emission factors as described by Koffi et al. (2017). Signatories can choose to use standard emission factors from Intergovernmental Panel on Climate Change (IPCC) or emissions factors which include Life Cycle Assessment (LCA). This is true also for biomass and biofuels which can therefore be assumed carbon neutral or not or include emissions as calculated through LCA.

Another important point to underline is that the results presented in this analysis, in terms of BEI and MEI activity and emissions data, are aggregated to the macro-sector level, whereas in the baseline and progress reports data is allocated in the due sub-sector (e.g. municipal transport). Therefore, in this study, whenever there is a reference to the transport or the residential macro-sectors it is always implied that the reference is to the sectors and carriers explicitly reported by the city within that macro-sector (and not the complete macro-sector).

Furthermore, experience has shown that not all the data collected on the CoM platform can be considered complete and reliable. This is because of the voluntary nature of the initiative, the difficulty of adapting sometimes local specificities to the CoM reporting framework, and the occurrence of inputting errors. For these reasons, in order to build a robust and reliable sample of CO_2_ emission inventories a series of checks is developed to identify and remove the errors. These checks are part of the validation procedure that the Joint Research Centre (JRC) performs on the data, subsequently providing to the cities. JRC has observed that these feedback cycles improve the quality of data reported over time. That is particularly important in this analysis, as we are considering the effect of progress reported in the emission inventories.

Details on this validation procedure and further checks that are carried out specifically for this work are described in the Supplementary Information (SI).

**Fig. 2:**
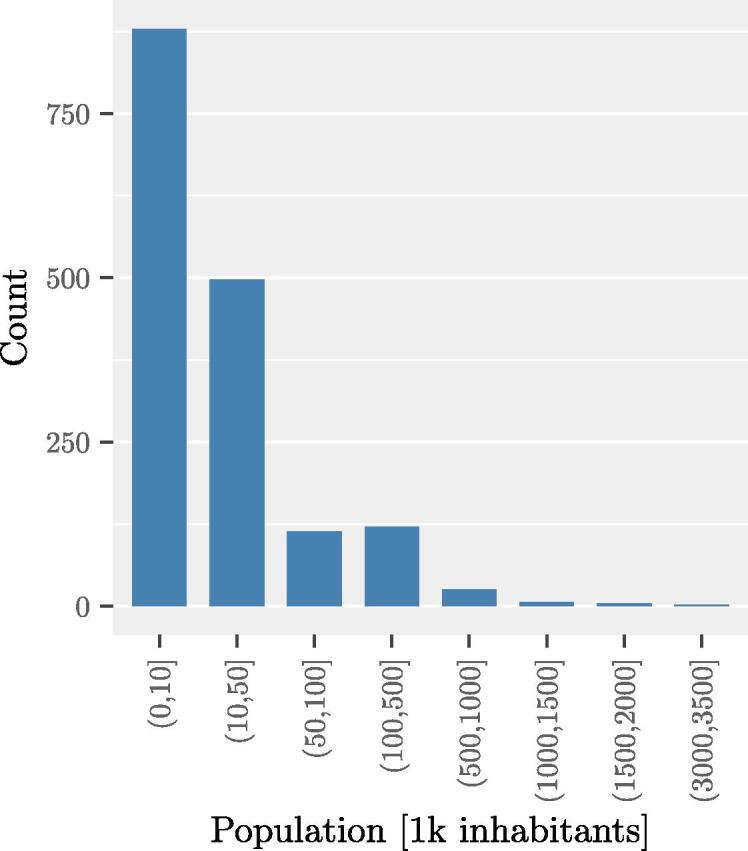
Number of signatories per class of inhabitants.

Finally, the analysis concerns 1653 signatories whose inventories were selected in terms of quality and completeness, out of the 1845 signatories of the initial data-set. As shown in Fig. 2, most of the signatories have less than 50000 inhabitants. Signatories range from communities of 53 inhabitants to cities and agglomerations of over 3 M inhabitants. The total population considered amounts to about 84 Million inhabitants for the BEI and about 88 M inhabitants for the MEI, with a median of about 9 k inhabitants, and an average of 50 k inhabitants.

**Fig. 3:**
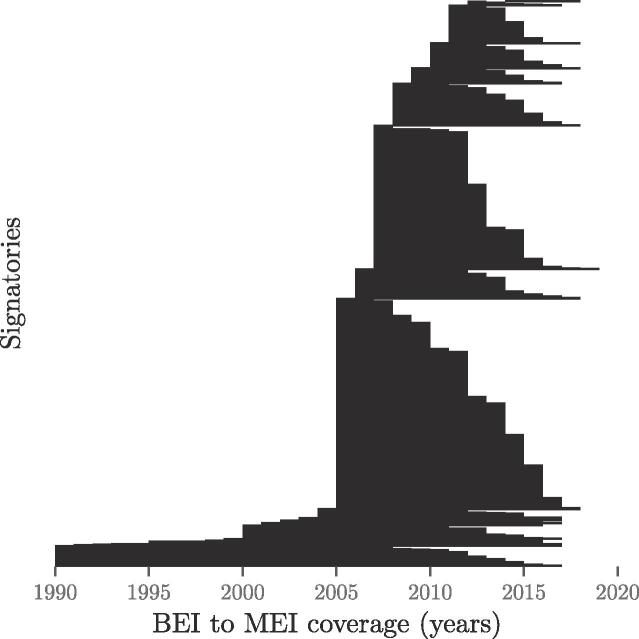
Variability of reporting years. BEI year and corresponding interval to the MEI for each signatory.

As said earlier, signatories are free to chose their reporting years, which are summarised in Fig. 3. This figure displays the variability of the BEI as well as the interval between the BEI and the MEI (which ranges between 1 and over 25 years). This variability makes the comparison between signatories difficult and should therefore be taken into consideration in the interpretation of results.

### 2.2 CoM - GAINS matching: estimate of air pollutant emission factors

The estimate of air pollutant emission factors is based on the detailed matching between CoM sectors and carriers to the corresponding GAINS ones. Details of the matching, the GAINS scenario considered, and corresponding calculations of emission factors (depending on the country and year) are reported in the SI.

The main simplifying assumptions underlying these estimates are that the national emission factors are assumed representative of the local ones in each city and that emission factors vary linearly within 5 years intervals (as this is the data available from GAINS). The first assumption is a very uncertain and simplifying one, that does not allow to evaluate measures specifically affecting the emission factors at the local level. In these cases only the effects due to the variation of activity can be accounted for. Examples of measures that can affect the emission factors are: a specific type of new natural gas bus substituting part of the public transport fleet, specific systems for biomass use in the residential sector, etc. The difference between national average and local emission factors can be especially significant for larger cities that may implement additional regulations with respect to the national ones, as for example a low emission zone (banning older and most polluting vehicles from a certain area) or a ban on the burning of wood or other fuels. The analysis could therefore be greatly improved by the integration of local data. Larger cities in particular may have more detailed information about their locally relevant emission factors. Most of the signatories are however represented by small to medium size cities, as shown in Fig. 2, therefore, integrating local data may change results for single cities but the same overall trends can be expected. At this stage, the analysis presented in this study provides a simple and consistent approach to evaluate signatories across Europe, taking at least into account national differences.

In the analysis two sets of air pollutant emission factors are used. A first set considers emission factors for the BEI and the MEI corresponding to their respective years. The evolution of emission factors through the years is taken into account and this set is referred to as *With Technological Improvement*. A second set considers a hypothetical world without technological improvement, that is to say both BEI and MEI use the same emission factors corresponding to the year of the BEI (in practice this means that, in this scenario, European regulations impact is not considered). In this case, the changes in fuel mix and consumption levels are considered but not the evolution of the technologies. This set is referred to as *Without Technological Improvement*. CO_2_ emission factors are taken as reported by the cities in the CoM for both the BEI and the MEI. This implies that ‘Technological Improvement’ refers only to local emissions of air pollutants and not to CO_2_ emissions.

In the SI the description of the methodology using EDGAR (Crippa et al., 2018) (instead of GAINS) is also provided, as well as in the code. EDGAR is currently being updated, therefore, the methodology and the results presented refer to GAINS. A preliminary analysis using EDGAR shows the same trends and general results. However, results for a single city may vary considerably as they strongly depend on the emission factors, which are calculated with different approaches in the two inventories.

## 3 Estimated pollutants emission changes per city and sector

**Fig. 4:**
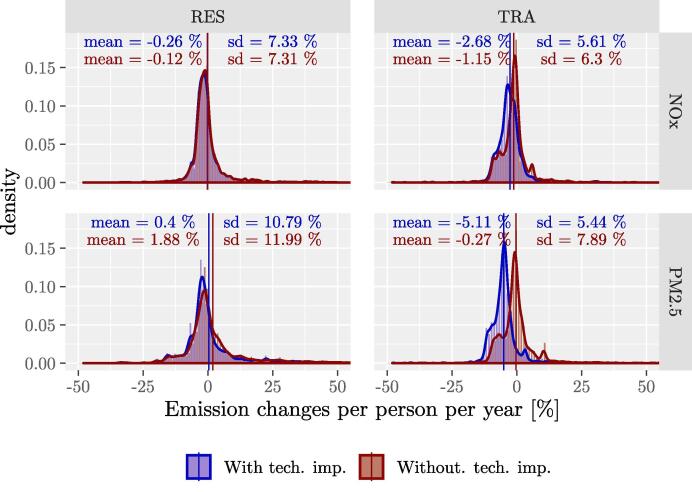
Air pollutant emission changes density plot. Percentage changes of emissions of air pollutants per person per year as estimated for each signatory. The top row represents NOx emissions changes in the residential (RES) and transport (TRA) respectively. The bottom one represents PM2.5 emission changes again in the transport and residential sectors respectively. The curves in blue represent changes considering technological improvement, the red ones represent changes without technological improvement (that is, the air pollutant emission factors for the MEI are the same ones as used for the BEI). Mean and standard deviation (sd) are calculated considering only data shown in the plots (i.e. outliers are removed, the data shown represents 96% of the selected signatories).

As described in Section 2, signatories report, for a baseline year of their choice and for a monitoring year, their CO_2_ emissions in a BEI and a MEI respectively. In this study we estimate the corresponding air pollutant emissions and we are especially interested in the changes between the two years. Results in terms of air pollutant emission changes per pollutant (PM2.5 and NOx) and (macro) sector (the residential sector and the transport one, referred to in the figures hereafter as RES and TRA), are summarised in Fig. 4. Results are reported per year assuming a linear change between emissions in the BEI and MEI years. Both the cases ‘with’ and ‘without’ technological improvement are presented.

Fig. 4 shows that the signatories considered (removing outliers, thus considering 96% of the selected signatories) display on average a reduction of air pollutant emissions, apart from PM2.5 emissions in the residential sector, which displays an average increase. Nevertheless, a large variation between cities can be observed. Technological improvement always reduces the mean emissions reported by cities, with the biggest effect in the case of PM2.5 in the transport sector. The average values are however sensitive to outliers, median values (not reported) both considering and not considering outliers show the same trends but always correspond to a reduction (including for PM2.5 emissions in the residential sector).

**Fig. 5:**
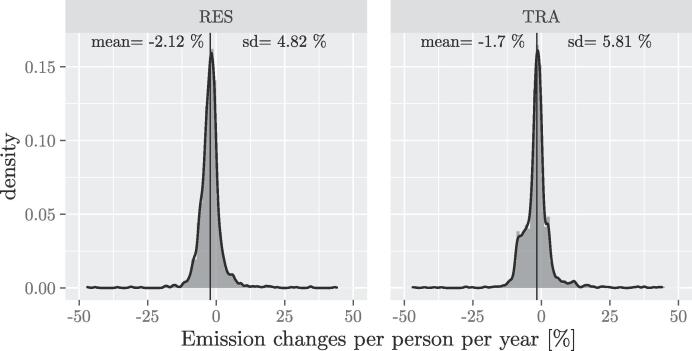
CO_2_ emission changes density plot. Percentage changes of CO_2_ emissions per person per year, as reported by each signatory for the residential (RES) sector and the transport (TRA) one. Mean and standard deviation (sd) are calculated considering only data shown in the plots (i.e. outliers are removed, the data shown represents 96% of the selected signatories).

Overall results for the corresponding CO_2_ emission changes per person per year, are reported in Fig. 5. The mean values show a reduction of emissions for both the residential sector and the transport one, in this case means and medians are very similar. It should be noted that these emission changes refer to an intermediate monitoring year and not the final target. Furthermore, even though they depend on the sample of cities considered, they are consistent with the results reported in Bertoldi et al. (2019).

In the following paragraphs, results are further elaborated. In order to better understand trade-offs and co-benefits between CO_2_ and air pollutant emissions.

## 4 CO_2_ versus air pollutant emissions: co-benefits and trade-offs

**Fig. 6:**
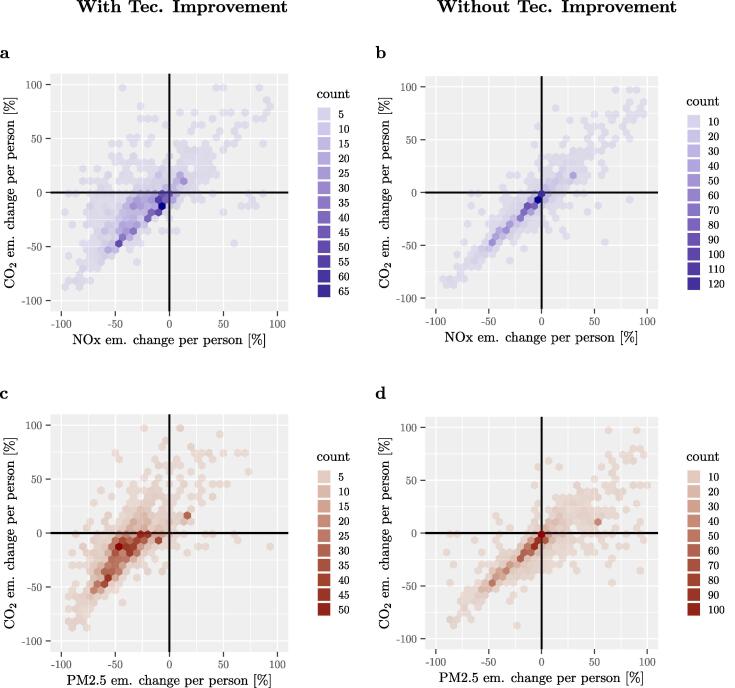
Count plot of CO_2_ vs air pollutant emission percentage changes (per person) for the transport sector. Changes are reported per person with respect to the BEI. Figures on the left represent changes with technological improvement, figures on the right represent changes without (that is the air pollutant emission factors for the MEI are the same one used for the BEI). The color shades represent the number of data-points found in each hexagon. (a) and (b) refer to CO_2_ vs NOx emissions (with and without technological improvement respectively). (c) and (d) refer to CO_2_ vs PM2.5 emissions (with and without technological improvement respectively).

It is interesting to present results in terms of relative changes per person between the MEI and BEI years, directly showing what signatories report in terms of CO_2_ emissions versus the ones estimated in this study in terms of air pollutant emissions. This allows to clearly trace the progress of each signatory and identify trade-offs and co-benefits. Fig. 6 displays results considering only the transport sector (TRA) and pollutant dimensions in the case ‘with’ technological improvement (left side of the figure) and ‘without’ it (right side of the figure). Rather than representing each result for each signatory, a count plot is presented. Fig. 6 visually shows the effect of technological improvement as points clearly move to the right (i.e. more air pollutant emissions for the same CO_2_ emissions) in the case without technological improvement. For the residential sector the effect is not that pronounced and the corresponding analysis is presented in the SI. Furthermore it should be noted that, for representation purposes, the axis are limited and not all data-points are represented. Outliers are, in particular, very high percentage changes of PM2.5 emissions per person in the residential sector (see SI). These changes usually correspond to very low emission levels in the BEI, which increase considerably, for example, because of the rise of biomass burning, in the MEI. It should be underlined that emission factors for biomass burning are extremely uncertain.

**Fig. 7:**
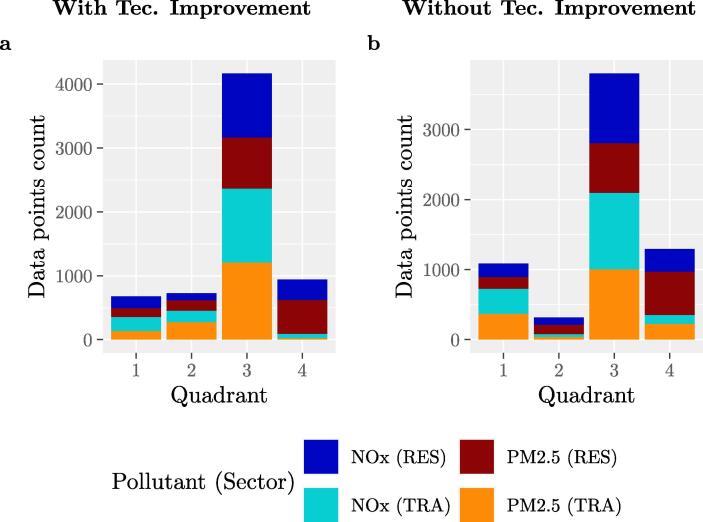
Number of data points in each quadrant. Number of data-points for all pollutant-sector combinations of the corresponding panels (with and without technological improvement) of Fig. 6 for the transport sector and the corresponding figure for the residential sector (in the SI). (a) represents the counts with technological improvement, (b) represents the counts without technological improvement.

To better show the changes between the two panels, Fig. 7 displays the number of data-points in each quadrant, including both the RES and TRA sector. For each sub-figure in Fig. 6, the 1st, 2nd, 3rd and 4th quadrants refer to the top-right, top-left, bottom-left and bottom-right of the graph respectively.

Analysing Fig. 6 for the transport sector, the corresponding figure for the residential one (in the SI) and Fig. 7 summarising all results, it is possible to make the following statements.

Starting from the case ‘With Technological Improvement’:

• Most results are in the 3rd quadrant (bottom-left), showing both reduction of emissions of CO_2_ and air pollutants between the BEI and the MEI and therefore co-benefits between air quality and climate change. Measures that bring cities to this quadrant are the ones that lead to reduction of activity (such as stimulating walking, cycling, energy efficiency measures, etc.) as well as the ones that lead to a reduction of emission factors (such as clean public transport, scrapping of old cars etc.) but only when associated with a reduction of activity. Energy efficiency measures applied in the context of the CoM are analysed in detail in Monforti-Ferrario et al. (2018).
• The 4th quadrant (bottom-right) represents the trade-off between a decrease of CO_2_ emissions and an increase of air pollutant emissions. Most of the points in this quadrant correspond to PM2.5 emissions in the residential sector.
• The 1st (top-right) and 2nd quadrants (top-left) have the smallest number of points. The 1st quadrant represents a detrimental effect for both types of emissions that can be attributed to increased activities. The 2nd quadrant represents a reduction of air pollutant emissions despite an increase of CO_2_ emissions. This is mainly due to increased activities which can, in certain cases, be compensated by a reduction of air pollutant emission factors due to technological improvement.

In order to evaluate the role of technological improvement, results can be compared to the cases ‘Without Technological Improvement’:

• As expected, results are shifted to the right, with more emissions of air pollutants (the emissions of CO_2_ are unchanged). This shows the importance of the technological improvement.
• Many points that are in the 2nd quadrant in the ‘With Technological Improvement’ case shift 1st quadrant in the ‘Without Technological Improvement’, these are especially points in the transport sector. This is true also for some data points in the 3rd quadrant that are shifted to the 4th one. In these cases the air pollutant emission reductions would have not been possible without technological improvement.

### 4.1 Trade-offs in the residential sector: biomass and PM2.5 emissions

**Fig. 8:**
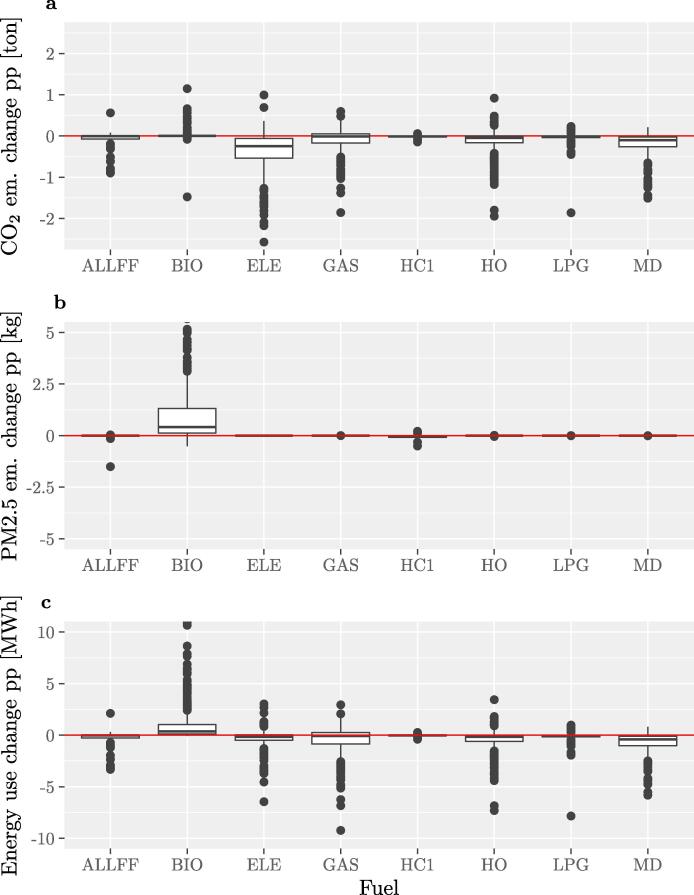
Explaining trade-offs in the residential sector: emissions and energy use changes (per person) per fuel. Data-points falling in the 4th quadrant for PM2.5 emission (see Fig. 7) in the case without technological improvement are presented considering CO_2_ emissions changes (a), corresponding PM2.5 emission changes (b), and energy use changes (c) with respect to the carriers (only the main carriers are displayed). As explained in the main text, district heating and cooling networks are not accounted for, whereas for electricity only CO_2_ emissions are considered as we do not know where and how the electricity is being produced.

It is interesting to analyse more in detail the 4th quadrant where, as shown in Fig. 7, most points refer to PM2.5 emissions in the residential sector, and correspond to reductions in CO_2_ with an increase of air pollutant emissions. These points are analyzed in Fig. 8 in terms of emission changes (PM2.5 and CO_2_) and energy use change per person and per fuel (see the SI for the complete list of acronyms). For most cities in this quadrant CO_2_ emission changes are due to reduced fuel use (especially electricity (ELE), heating oil (HO), diesel (MD)) along with an increase of the use of biomass (BIO) (see top and bottom panels). The middle panel shows that, in absolute terms, BIO contributes to the largest increases in PM2.5 emissions.

A note of caution: with the data available we cannot know if, for example, some cities decided to report biomass use with greater detail in the MEI with respect to the BEI. For example, sometimes, the reported energy use for biomass in the BEI can be zero or close to zero, whereas it increases by many folds in the MEI. For biomass, this has little impacts on CO_2_, but it can have a very large impact on air pollutant emissions (this may be the reason for the outliers mentioned above). Furthermore, as underlined earlier, we do not know which biomass technologies the city implemented, therefore, at this stage we have to rely on national average emission factors.

### 4.2 Trade-offs in the transport sector: the importance of technological improvement

Another interesting quadrant is the 2nd one (top-left in Fig. 6), where most of the points belong to the transport sector for the case with technological improvement (see Fig. 7). Many of these points shift to the 1st quadrant in the case without technological improvement. The points that shift represent the signatories that, thanks to technological improvement, obtained a reduction of air pollutant emissions despite an increase in CO_2_ emissions.

**Fig. 9:**
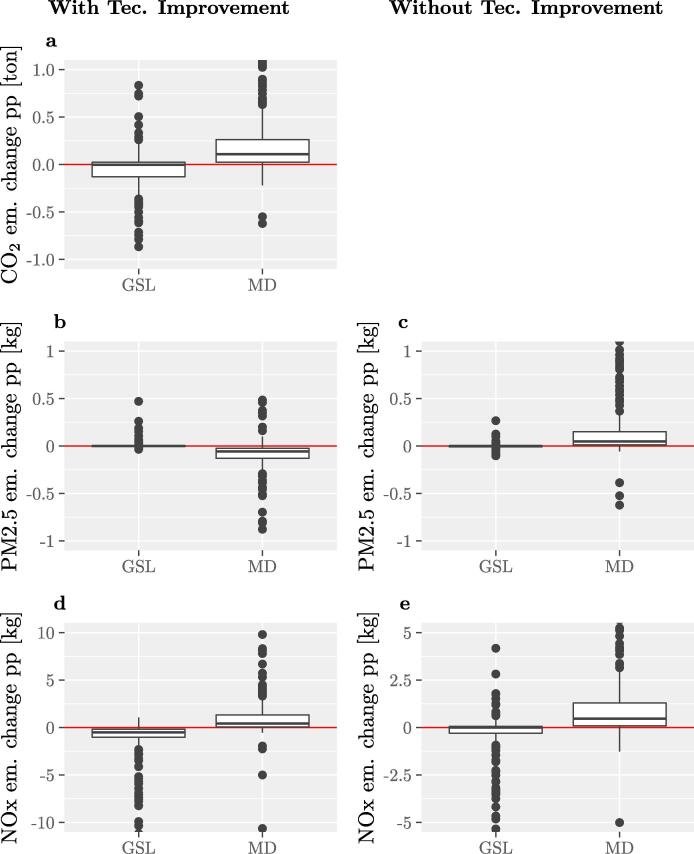
Explaining the effect of technological improvement in the transport sector: emissions changes (per person) per fuel. CO_2_ (a), PM2.5 (b, c) and NOx (d, e) emission changes (per person) in the transport sector. The points considered are only the ones that move from the 2nd quadrant of Fig. 6 in the case with technological improvement (higher CO_2_ but lower air pollutant emissions) to the 1st quadrant in the case without technological improvement (higher CO_2_ and higher air pollutant emissions). Figures on the left (c, b, d) represent these points in the case with technological improvement and figures on the right (c, e) represent them in the case without it. To note: CO_2_ emissions always refer to what is directly reported by the signatories.

To better explain the changes between BEI and MEI for these signatories, Fig. 9 shows the ‘carrier’ detail for the points of the transport sector shifting from the 2nd to the 1st quadrant. Only diesel (MD) and gasoline (GSL) are displayed here. This figure shows that the use of MD and consequently related CO_2_ emissions have generally increased between the BEI and the MEI years, whereas GSL use spans across the zero line with both increases and reductions. This means that, for these signatories, a switch from GSL to MD did not bring benefits in terms of overall CO_2_ emissions from TRA, on the contrary, emissions increased. This increase can be due to an increase of fuel consumption, due for example to growing distance travelled or larger vehicles, which is not compensated by the efficiency gain attributed to diesel vehicles.

It is interesting to see how the increase of MD use, considering technological improvement, is generally associated to a reduction in PM2.5 emissions and an increase in NOx emissions (we are using updated NOx emission factors for diesel, coherent with the most recent (2019) available data, already considering real driving emissions). Without technological improvement there would have been an increase of PM2.5 emissions and a higher increase in NOx emissions. The decrease in NOx emissions associated to GSL is due both to the decrease in activity level and the technological improvement.

## 5 Conclusion

The methodology developed in this study allows to link climate mitigation to air quality, consistently and considering local data. This link is rarely considered at the local scale, which means that data for validation of our approach are scarce and patchy. In any case, we performed a first attempt to crosscheck our approach, comparing results for single cities to the air pollutant emissions that the cities report elsewhere (e.g. in their air quality plans or their own scenarios). Preliminary results can be found in the SI.

The approach also has some limitations, already highlighted in the text or in the relevant sections in the SI but that the authors find useful to also underline and summarise here. (i) The main one is that, at this stage, we only use national emission factors. We cannot therefore take into account a local technology mix which may be different from the national one (e.g. the specific composition of the vehicle fleet in terms of Euro-standards in a city). (ii) One should also keep in mind that the CoM is not designed to tackle air quality in the first place, and any estimate of air pollutant emissions is only a rough approximation which is not meant to substitute a detailed air quality plan. As a consequence we cannot account for all local emissions of air pollutants, as for example air pollutant emissions due to local energy generation (local electricity or heat and cold generation in a district heating/cooling network). District heating networks, in particular, should be evaluated in more detail. For example, trade-offs between climate and air quality can arise when the heat source is switched from large industries and power plants outside the city to smaller biomass fueled installations in or near the city itself. (iii) Furthermore, it should be underlined that the approach, given the data available, does not consider single measures but rather the ‘bundle of measures’ implemented by signatories together with the effect of national policies. It is therefore not possible to ascribe the changes in emissions of air pollutants entirely to the CoM initiative. (iv) Finally and obviously, the quality of results strongly depends on the quality and detail of the input data submitted by the signatories (see SI for details on data-cleaning).

Taking into account these limitations, the methodology developed in this work and applied to the inventories of 1653 selected signatories, allows drawing the following conclusions:

• The approach allows to clearly identify trade-offs and co-benefits obtained by signatories in terms of air pollutant emissions and CO_2_ emissions between baseline and monitoring years.
• Technological improvement has been of fundamental importance to limit the increase of air pollutant emissions despite increases in consumption patterns. This is especially true for PM2.5 emissions in the transport sector (in particular for diesel cars).
• Most of the changes reported by the cities correspond to co-benefits for both climate change mitigation and air quality related emission reductions.
• It is possible to clearly identify trade-offs between air quality and climate, as for example due to the use of biomass in the residential sector (reducing CO_2_ and increasing PM2.5 emissions).
• We can also identify changes that are shown to be detrimental for both air quality and climate, associated to increases in energy consumption.

The approach uses, at the moment, only information the signatories already provide to the CoM but it could be greatly improved if local information on air pollutant emission factors were available. This would allow cities to better take into consideration their local conditions as well as include emissions for local energy production, such as district heating/cooling networks and local electricity generation. Furthermore, results show the importance of the accuracy and detail of this large and unique data-set, which can provide useful insights beyond climate change mitigation.

Further work will be devoted to extend this methodology, to assess impacts on air quality and health (and not only emissions) and to allow signatories to evaluate themselves the impact of single actions and measures (rather than their overall progress). Finally the aim of this work, beyond the analysis presented in this paper, would be to allow cities themselves to evaluate the impact on air quality of their local climate mitigation measures, to limit possible trade-offs between these two objectives.

## 6 Code availability

The code, in the R language (R Core Team, 2017), developed for the analysis presented in this study with example input data are available at: https://github.com/esperluette/airpollutants_script_for_com.

## CRediT authorship contribution statement

**Emanuela Peduzzi:** Conceptualization, Methodology, Investigation, Formal analysis, Data curation, Validation, Visualization, Writing - review & editing, Supervision. **Marta Giulia Bald:** Data curation, Methodology. **Enrico Pisoni:** Conceptualization, Methodology, Writing - review & editing. **Albana Kona:** Data curation, Methodology. **Paolo Bertoldi:** Data curation, Writing - review & editing. **Fabio Monforti-Ferrario:** Methodology, Writing - review & editing, Project administration.

## Declaration of Competing Interest

The authors declare that they have no known competing financial interests or personal relationships that could have appeared to influence the work reported in this paper.
