# Supplementary material for: Impacts of a climate change initiative on air pollutant emissions: Insights from the Covenant of Mayors
Source: Environ Int. 2020 Dec;145:106029. doi: 10.1016/j.envint.2020.106029 (PMC7569603; doi:10.1016/j.envint.2020.106029)
Supplement: Supplementary data 1 [file mmc1.pdf]

Supplementary Information - Impacts of a climate change initiative on air pollutant emissions: insights from the Covenant of Mayors

Emanuela Peduzzi[a,*], Marta Giulia Baldi[a], Enrico Pisoni[a], Albana Kona[a], Paolo Bertoldi[a], Fabio Monforti-Ferrario[a]

[a]*European Commission, Joint Research Centre, Via Enrico Fermi 2749, 21027 Ispra, VA, Italy*

CoM-GAINS sector and carrier matching

In this section the detail of the matching of sector and carriers between Covenant of Mayors (CoM) and Greenhouse Gas - Air Pollution Interactions and Synergies (GAINS) and further detail on the calculation of emission factors are reported in detail.

Table a reports the matching of the CoM sectors to the GAINS ones. Unfortunately, a one to one match is not possible. All CoM sectors referring to buildings are mapped to the domestic (DOM) sector of GAINS (including residential, commercial and services buildings), the ones relative to transport are mapped into several GAINS sector.

The matching of carriers and fuels is more straightforward and is reported in Table 2. Most fuels have a one to one match, the exceptions are biofuels and other biomass matched to the category BIO, other fossil fuels matched to the category ALL_FF, (standing for 'all fossil fuels)', heating oil matched to the fuel HO (heating oil). BIO, ALL_FF and HO are not GAINS fuels. HO is associated for the domestic sector to the HF (heavy fuels) and MD (medium distillates) reported in GAINS. Some cities however report the use of HO in the transport sector, which could be an input error. Because there is not HO matched to transport in GAINS, in this case, the assumption is that they are actually referring to MD.

BIO, when used in the residential sector, corresponds to all biogenic fuels used in this sector in the selected scenario (denoted as OS1 or the specific fuel name in the GAINS database, see Table 3). On the contrary, when used in the transport sector it is matched to all fuels used in this sector (as the scenario does not report biofuels as a separate fuel). In this case, in the absence of more detailed data, the assumption is that the emission factors of air pollutants of biofuels and bio-fossil fuels mixtures are similar to those of the corresponding fuel mixture reported in the scenario. This is a limitation due to the emission factors we have available at the moment through the GAINS database. The matching could be refined and improved

*Corresponding Author: emanuela.peduzzi@ec.europa.eu

CoM sector	GAINS sector
municipal buildings equipment facilities	DOM
tertiary buildings equipment facilities	DOM
residential buildings	DOM
buildings equipment facilities non allocated	DOM
buildings equipment facilities sector	DOM
municipal fleet	TRA_RD_LD4T, TRA_RD_LD4C
public transport	TRA_RD_HDB
private and commercial transport	TRA_RD_LD4C, TRA_RD_LD4T
transport non allocated	TRA_RD_LD4C, TRA_RD_LD4T, TRA_RD_HDB
transport sector	TRA_RD_LD4C, TRA_RD_LD4T, TRA_RD_HDB

Supplementary Table 1: **Matching of sectors**. Matching of sectors as defined by the CoM to the ones of the GAINS database.

if more detailed emission factors were available, e.g. for biofuels or plant oil for the different sub-sectors of transport. However, it should be underlined that, in the current CoM database, the reporting of biogenic fuels in the transport sector is not always consistent and detailed, therefore the assumption used in this study can be considered reasonable.

According to the report Koffi et al. (2017), other fossil fuels should correspond to peat and the fossil fraction of municipal solid waste. However, looking at the data, this carrier is sometimes associated to the transport sector as well as the residential sector. In this analysis, 'other fossil fuels', is associated to ALL_FF used in the corresponding sector under the assumption that the reported energy use is correct.

The GAINS on-line platform provides emissions for different pollutants and activities, for each of its sectors fuels combinations, at 5 year intervals. From this data the emission factors for the CoM sector and carrier combinations are estimated. The emission factors are linearly interpolated between the scenario years closest to the inventory (Baseline Emission Inventory (BEI) or Monitoring Emission Inventory (MEI)) year.

The scenario considered for estimating the implied emission factors is the 'REF_post2014_CLE' (id: SC11_CLE2017F) (Amann et al. (2011); International Institute for Applied Systems Analysis (IIASA) (????)). This is the reference scenario including legislation introduced after 2014

**CoM carrier**	**GAINS fuel**
electricity	ELE
natural gas	GAS
liquid gas	LPG
heating oil	*HO*
diesel	MD
gasoline	GSL
lignite	BC1
coal	HC1
other fossil fuel	*ALL FF*
plant oil	*BIO*
biofuel	*BIO*
other biomass	*BIO*

Supplementary Table 2: **Matching of carriers**. Matching of carriers as defined by the CoM to the fuels defined in the GAINS model, fuels displayed in *italic* are not part of the GAINS database.

(the 'post-2014' legislation). The energy scenario is based on the PRIMES 2016 REFERENCE with adjustments needed to reflect impacts of ECODESIGN Directive.

CoM-EDGAR sector and carrier matching

The same analysis presented in this study can be carried out using the Emission Database for Global Atmospheric Research (EDGAR) database. In this case the emission factors are not the result of a scenario and a model, but rather the result of the careful compilation of emissions, activities as well as abatement technologies around the world (Crippa et al. (2018)). The EDGAR database is currently being updated, therefore we preferred presenting results in the main text of this work using the GAINS model. However, a preliminary analysis on the same CoM dataset and using both inventories, shows comparable results and the same trends. We provide therefore also the code for using EDGAR data. For completeness, Table a and Table 5 report CoM and EDGAR matching for sectors and carriers. Table 6 reports the description of the EDGAR sectors and fuels.

Abbreviation	Description
DOM	Residential, commercial, services, agriculture, etc.
TRA_RD_HDB	Heavy duty vehicles - buses
TRA_RD_LD4C	Light duty vehicles: cars and small buses with 4-stroke engines
TRA_RD_LD4T	Light duty vehicles: light commercial trucks with 4-stroke engines
BC1	Brown coal/lignite, grade 1
ELE	Electricity
GAS	Natural gas (incl. CNG and derived gases)
GSL	Gasoline and other light fractions of oil; includes biofuels
HC1	Hard coal, grade 1
HF	Heavy fuel oil
LPG	Liquefied petroleum gas
MD	Medium distillates (diesel, light fuel oil; includes biofuels)
OS1	Biomass fuels
–ARD	Agricultural residuals - direct use
–BGS	Bagasse
–BIO	Biogas
–BMG	Biomass gasification
–CHCOA	Charcoal
–DNG	Dung
–FWD	Fuelwood direct

Supplementary Table 3: **Description of the GAINS database labels.**

CoM sector	EDGAR sector
municipal buildings equipment facilities	RCO
tertiary buildings equipment facilities	RCO.COM
residential buildings	RCO.RES
buildings equipment facilities non allocated	RCO.OTH
buildings equipment facilities sector	RCO
municipal fleet	TRO.PC0+LD0
public transport	TRO.BS0
private and commercial transport	TRO.PC0+LD0
transport non allocated	TRO.BS0+PC0+LD0
transport sector	TRO.BS0+PC0+LD0

Supplementary Table 4: **Matching of sectors for the EDGAR database.**

CoM carrier	EDGAR fuel
electricity	ELE
natural gas	NGS
liquid gas	LPG
heating oil	fossil OIL
diesel	DIE
gasoline	MOG
lignite	LGN+BRC
coal	COAL
other fossil fuel	all fossil
plant oil	BIO
biofuel	BIO
other biomass	BIO

Supplementary Table 5: **Matching of carriers for the EDGAR database.**

Abbreviation	Description
RCO	The complete Residential sector
RCO.COM	Commercial/institutional
RCO.OTH	All remaining from fuel combustion (residential)
TRO.ROA.BS0	Buses (combustion and non-exhaust)
TRO.ROA.LD0	Light Duty (combustion and non-exhaust)
TRO.ROA.PC0	Passenger Cars (combustion and non-exhaust)
all fossil	all excluding BIO
BIO	Biomass fuels
COAL	Solid fossil fuels
DIE	Gas/Diesel (Diesel)
fossil OIL	Fossil oil and oil products
LGN+BRC	Lignite/Brown Coal
LPG	Liquefied petroleum gases
MOG	Motor gasoline (petrol)
NGS	Natural Gas

Supplementary Table 6: **Description of the EDGAR database labels**

Data cleaning

The Joint Research Centre (JRC) carries out a validation procedure on the data submitted by the signatories. Part of this procedure, consists of a broad consistency check between the order of magnitude of reported emissions per capita in the BEI and MEI datasets. Values are compared to the average ones per Country from the EDGAR database. Data that are outside the validity boundaries (+/- 200% of national average emission factors compared to Intergovernmental Panel on Climate Change (IPCC) 2006 factors) in large urban areas (population greater than 100.000 inhabitants) are checked individually with the Sustainable Energy and Climate Action Plan (SECAP) document and corrected if inconsistencies are noted. In small and medium towns, the order of magnitude is checked and corrected directly. Errors often arise simply because of misinterpretation of units (ktCO_2 instead of tCO_2 or kWh/year instead of MWh/year).

Further cleaning of the CoM data-set is carried out in order to limit, as much as possible, input errors for the specific analysis in this work. The following checks and changes are conducted:

- CO_2 emissions associated with no energy consumption, or vice versa, energy consumption of a fossil fuel reported without CO_2 emissions (rare): the missing value (CO_2 emissions or energy consumption) is calculated using default emission factors.
- Coal used for transport: at the moment the corresponding emissions are not considered (even though they could correspond to rail transport).
- Heating oil used in transport: is assumed as an input mistake and is considered as diesel.

Internal consistency checks between the macro-sectors in the BEI and MEI data-sets are also performed. As we are looking at changes (between the BEI and the MEI years), the following outliers are removed for the display of data and analysis.

- Sectors (intended as total transport or domestic) for which energy consumption is reported to be 0 or almost 0 for the BEI or the MEI are removed.
- Sectors (intended as total transport or domestic) for which the relative energy consumption or CO_2 emissions varies between the BEI and the MEI by less than -90% and more than +500% are removed.

Finally, the data displayed in the figures corresponds to 1655 Signatories.

Trade-offs at city level

In order to show the variability between cities, Figure 1 presents CO_2 with respect to NOx and PM2.5 emission changes per city and per person. As in the main text, the data is presented with a count plot. In panel A, most cities belong to the 3rd quadrant (where both emissions of air pollutants and CO_2 are reduced). In panel B, the number of cities in the 1st and 4th quadrants increases, which means that, without technological improvement, these cities would have had increased their emissions of air pollutants. For representation purposes the axis are limited and not all data-points are represented (removing outliers).

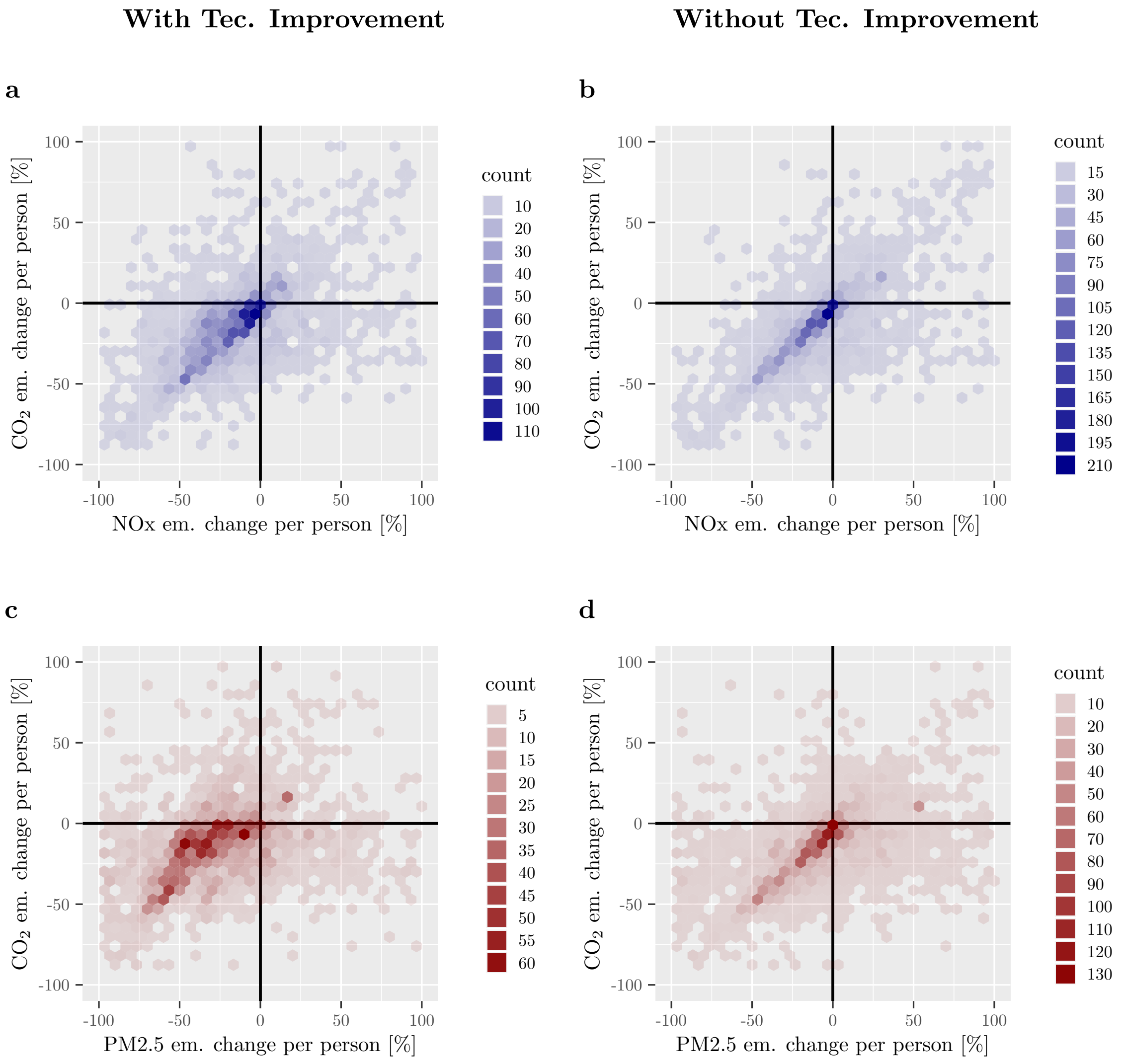

Supplementary Figure 1: **Count plot of CO_2 vs air pollutants emission percentage changes (per person) per signatory**. Changes are reported per person with respect to the BEI. (a) Represents changes with technological improvement, (b) changes without (that is the air pollutant emission factors for the MEI are the same one used for the BEI). The color shades represent the number of data-points found in each hexagon. (a) and (b) refer to CO_2 vs NOx emissions (with and without technological improvement respectively). (c) and (d) refer to CO_2 vs PM2.5 emissions (with and without technological improvement respectively).

Overall results

Figure 2 displays CO_2 emissions of the transport (TRA, circles) and domestic/residential (DOM, triangles) sectors as reported by each signatory versus the corresponding NOx (panel A) and PM2.5 (panel B) emissions as calculated in this study for both the BEI (filled markers) and their MEIs (empty markers) years. Emissions are reported considering the emission factors of the corresponding years, that is 'With technological improvement'.

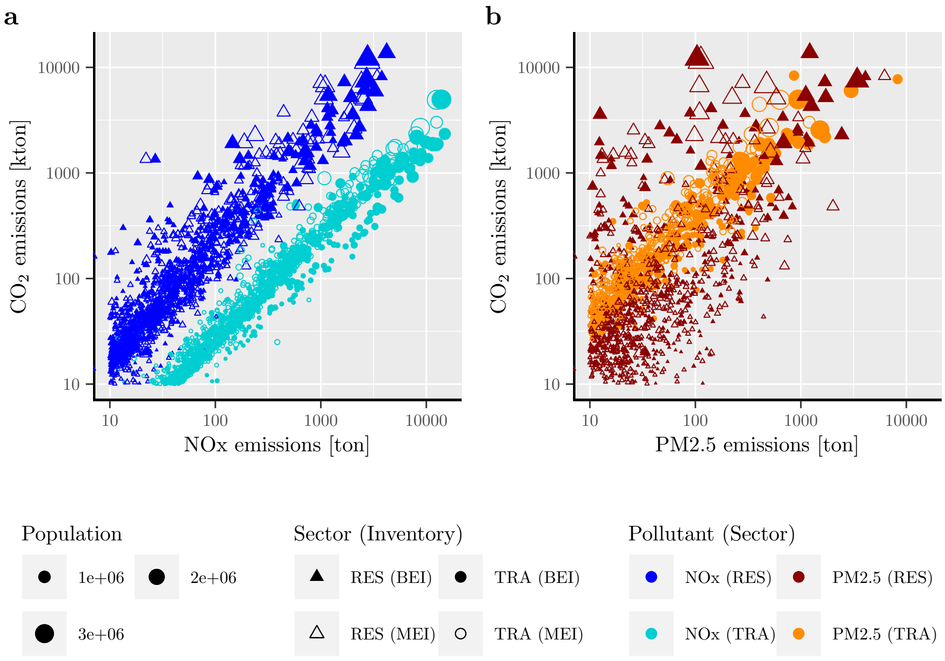

Supplementary Figure 2: **Absolute values of CO_2 vs air pollutants emissions** CO_2 emissions for the residential (DOM, triangles) and transport (TRA, circles) sectors as reported by the cities in their BEIs (filled markers) and their MEIs (empty markers) with respect to the NOx (a) and PM2.5 (b) emissions estimated in the current study. The size of the markers is proportional to the population of the city.

From Figure 2 t is evident that:

- For the same level of CO_2 emission, transport displays the highest NOx emissions.
- There is a clear correlation between air pollutant emissions and CO_2 emissions (as expected), The correlation is stronger for the transport sector (circles) whereas, for the residential one (triangles) and especially for PM2.5 (red triangles) data-points are more dispersed. This dispersion is due to the larger variability of fuels/technologies and therefore of emission factors used in the domestic sector.

Co-benefits and trade-offs for the residential sector

In the main text we presented the co-benefits and trade-offs for the transport sector, highlighting the effect of technological improvement. The corresponding results for the residential sector are presented in Figure 3. In this case the effect of technological improvement is less evident.

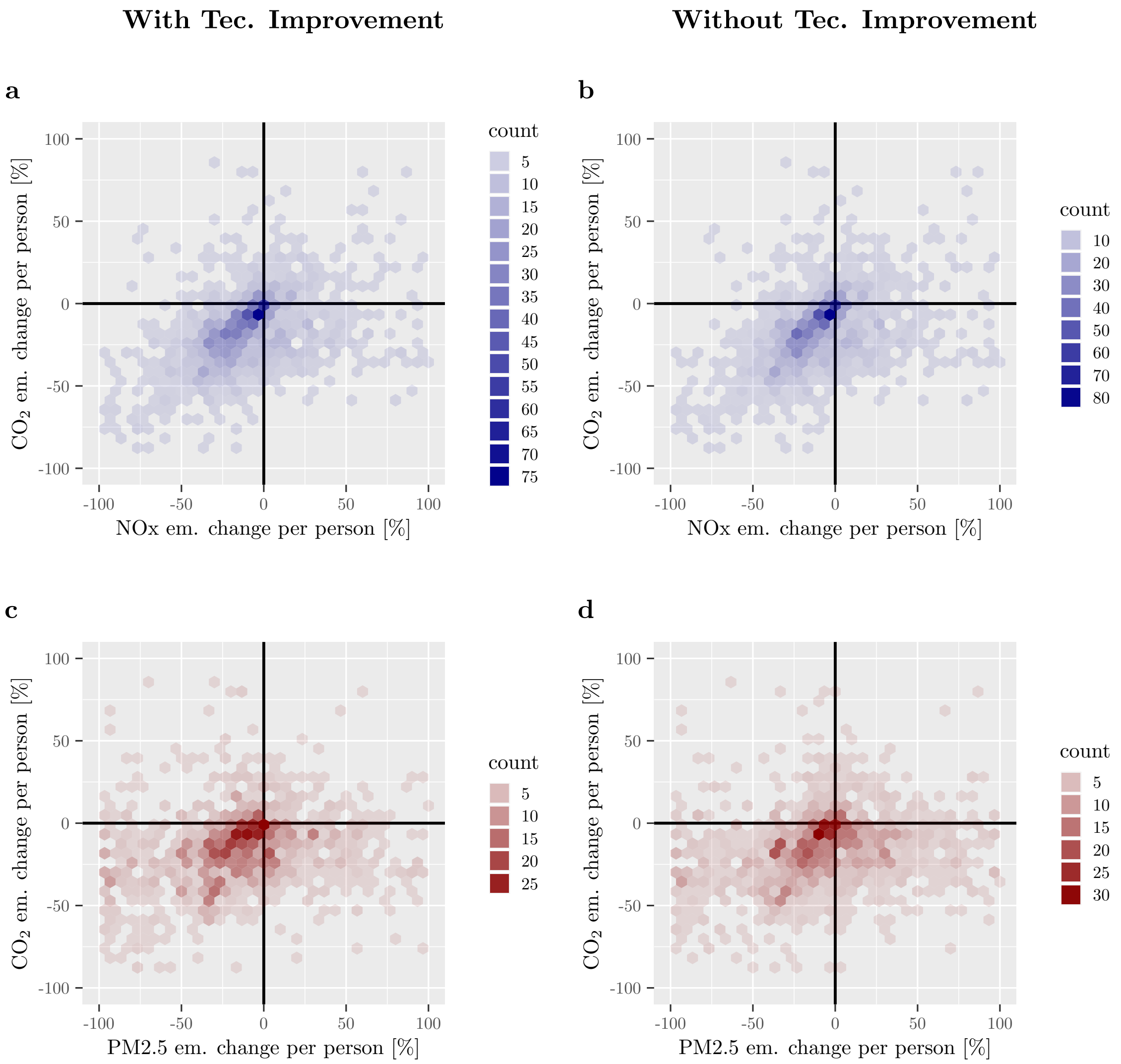

Supplementary Figure 3: **Count plot of CO_2 vs air pollutants emission percentage changes (per person) for the residential sector**. Changes are reported per person with respect to the BEI. Figures on the left represent changes with technological improvement, figures on the right represent changes without (that is the air pollutant emission factors for the MEI are the same one used for the BEI). The color shades represent the number of data-points found in each hexagon. (a) and (b) refer to CO_2 vs NOx emissions (with and without technological improvement respectively). (c) and (d) refer to CO_2 vs PM2.5 emissions (with and without technological improvement respectively).

1. Validation

A validation of this work was attempted by comparing the results obtained to the ones reported in the literature. This approach proved extremely difficult (and tedious). Literature values are obtained by looking at cities' air quality plans and local air pollutant emission inventories when available.

The results obtained for three cities, in their BEI and MEI years (or the closest available year) are reported in Figure 4.

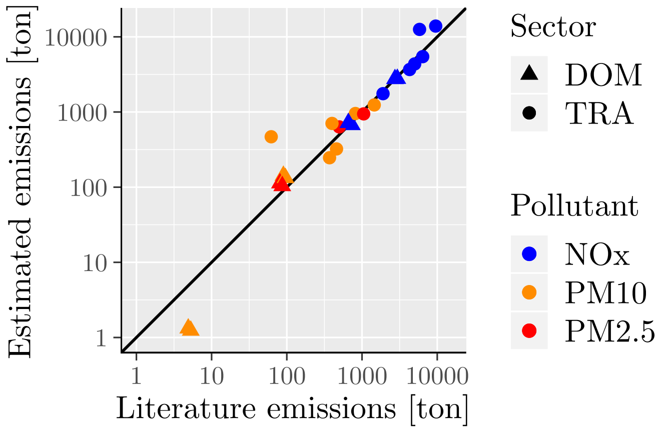

Supplementary Figure 4: Comparison of absolute emission values obtained in this work and the literature, for a selection of cities.

Some of the difficulties encountered during this comparison are, for example:

- It is not always possible to find data for the years corresponding to the BEI and the MEI years.
- CoM inventories generally report the emissions that the city itself, with its actions, can reduce. Therefore a one to one matching is not always possible.
- Following from the previous point, even at macrosector level (e.g. transport), literature data may have a different aggregation than the one provided by the CoM. E.g. it may include shipping or heavy duty transport.
- The boundaries of the city may also be different.

Given these constraints and limitations, it was not possible to carry out, at the moment, a more thorough comparison. The few results obtained, however, generally show a promising picture.

Code

The code supporting this analysis is available at:
`https://github.com/esperluette/airpollutants_script_for_com`

At the moment, the entire CoM database is not yet publicly available (though it is planned to become accessible soon) so it was not possible to provide the entire dataset used for this work. However, a fictive input data file is provided for one example city, which could allow any city to test the data they submit to the CoM with the proposed approach. Minimum input data is also provided from GAINS and EDGAR. Additional data from GAINS and EDGAR (i.e. for different countries and different years) can be accessed through their respective contacts and on-line platforms (see the code for more details).

References

Amann, M., Bertok, I., Borken-Kleefeld, J., Cofala, J., Heyes, C., Höglund-Isaksson, L., Klimont, Z., Nguyen, B., Posch, M., Rafaj, P., Sandler, R., Schöpp, W., Wagner, F., Winiwarter, W., 2011. Cost-effective control of air quality and greenhouse gases in Europe: Modeling and policy applications. Environmental Modelling & Software 26 (12), 1489–1501.

Crippa, M., Guizzardi, D., Muntean, M., Schaaf, E., Dentener, F., van Aardenne, J. A., Monni, S., Doering, U., Olivier, J. G. J., Pagliari, V., Janssens-Maenhout, G., 10 2018. Gridded emissions of air pollutants for the period 19702012 within EDGAR v4.3.2. Earth System Science Data 10 (4), 1987–2013.
URL `https://www.earth-syst-sci-data.net/10/1987/2018/`

International Institute for Applied Systems Analysis (IIASA), ???? GAINS Model.
URL `http://gains.iiasa.ac.at/models/index.html`

Koffi, B., Cerutti, A., Duerr, M., Iancu, A., Kona, A., Janssens-Maenhout, G., 2017. CoM Default Emission Factors for the Member States of the European Union - Version 2017. Tech. rep., European Commission, Joint Research Centre (JRC).
URL `http://data.europa.eu/89h/jrc-com-ef-comw-ef-2017`
